# Chloruretted Lotions in Confluent Variola

**Published:** 1844-07-27

**Authors:** 


### RECORD OF MEDICAL SCIENCE.

### CHLORURETTED LOTIONS IN CONFLUENT VARIOLA.

*Dr. Bailleul* strongly recommends the application of chloruretted lotions to the skin, throat and nasal passages, in severe cases of confluent small-pox. He is of opinion that they serve to decompose the purulent matter in the pustules, and thereby to counteract the tendency that often exists to cutaneous and pulmonary asphyxia, by neutralizing the noxious agency of the purulent matter on the cutaneous and mucous surfaces. The fever of resorption—or secondary fever, as it is usually called—is thus rendered much less formidable; and, moreover, the emanation of poisonous effluvia from the body is in a great measure arrested. Considered as local applications, the chloruretted lotions refresh and re-invigorate the vital properties of the skin, and tend also to promote the cicatrisation of the greyish-colored ulcers which line the bottom of the variolous pustules. The practice here recommended may be expected to be of especial utility in prisons, hospitals, on board ship, &c., where patients are apt to be crowded together, and the danger of infection is therefore to be most dreaded.—*Med. Chir. Rev.*

